# Formation of Gold Nanoparticle Self-Assembling Films in Various Polymer Matrices for SERS Substrates

**DOI:** 10.3390/ma15155197

**Published:** 2022-07-27

**Authors:** Ksenia A. Maleeva, Ilia E. Kaliya, Anton P. Tkach, Anton A. Babaev, Michail A. Baranov, Kevin Berwick, Tatiana S. Perova, Alexander V. Baranov, Kirill V. Bogdanov

**Affiliations:** 1 Center of Information Optical Technologies, ITMO University, 197101 Saint Petersburg, Russia; khnykina.kseniya@mail.ru (K.A.M.); kaliyailya2802@gmail.com (I.E.K.); toni.tkach95@gmail.com (A.P.T.); a.a.babaev@itmo.ru (A.A.B.); mbaranov@mail.ru (M.A.B.); a_v_baranov@yahoo.com (A.V.B.); 2 School of Electrical and Electronic Engineering, Technological University Dublin, D07 EWV4 Dublin, Ireland; kevin.berwick@tudublin.ie; 3 Department of Electronic and Electrical Engineering, Trinity College Dublin, The University of Dublin, D02 PN40 Dublin, Ireland

**Keywords:** SERS, self-assembling, polymer matrix, sensors

## Abstract

Surface-enhanced Raman spectroscopy (SERS) is regarded as a versatile tool for studying the composition and structure of matter. This work has studied the preparation of a SERS substrate based on a self-assembling plasmonic nanoparticle film (SPF) in a polymer matrix. Several synthesis parameters for the SPF are investigated, including the size of the particles making up the film and the concentration and type of the self-assembling agent. The result of testing systems with different characteristics is discussed using a model substance (pseudoisocyanin iodide). These models can be useful in the study of biology and chemistry. Research results contain the optimal parameters for SPF synthesis, maximizing the SERS signal. The optimal procedure for SPF assembly is determined and used for the synthesis of composite SPFs within different polymer matrices. SPF in a polymer matrix is necessary for the routine use of the SERS substrate for various types of analytes, including solid samples or those sensitive to contamination. Polystyrene, polyvinyl alcohol (PVA), and polyethylene are investigated to obtain a polymer matrix for SPF, and various methods of incorporating SPF into a polymer matrix are being explored. It is found that films with the best signal enhancement and reproducibility were obtained in polystyrene. The minimum detectable concentration for the SERS substrate obtained is equal to 10^−10^ M. We prepared a SERS substrate with an analytical enhancement factor of 2.7 × 10^4^, allowing an increase in the detection sensitivity of analyte solutions of five orders of magnitude.

## 1. Introduction

The phenomenon of Raman scattering was theoretically predicted and experimentally verified over 90 years ago [1,2]. Many improvements to the method have been discovered since. An interesting advance in Raman spectroscopy occurred with the discovery of surface-enhanced Raman scattering (SERS). SERS made it possible to record Raman spectra from analytes for which it was previously impossible, either due to the low intensity or the interfering effect of luminescence. The electromagnetic and chemical mechanisms of the SERS phenomenon have been discussed elsewhere [3]. Reviews are regularly published that contain descriptions of the SERS technique [4,5,6].

SERS is a powerful analytical tool for studying the structure and composition of materials. There are many applications of SERS substrates, as part of chemosensors [7,8,9], sensors for detecting toxins [10,11], water and food analysis [12,13], sensors sensitive to enantiomeric composition [14], biosensors [15,16,17], research of biological samples in vitro [18,19,20] and in vivo [21], and also to study the mechanism of chemical reactions [22]. However, despite a large research effort on the development of SERS substrates, there is still interest in improving their efficiency and reproducibility so they can be used routinely for quantitative measurements. It is desirable to make a SERS substrate that can be used for different analytes or analyte types. In addition, it would be attractive to have the ability to carry out quantitative and structural studies at the same time. There are two types of enhancing substrates, viz., colloidal solutions, and solid substrates. The main disadvantage of colloidal-enhancing substrates is the time dependence of the enhancement efficiency due to the aggregation of colloids. Solid substrates do not suffer from this problem. Regularly patterned surfaces obtained via lithographic techniques [23], using the mask technique [24], thermal evaporation [25,26], or thermophoretically driven Au aerosol deposition [27] can solve problems with the reproducibility of surface distributions. There are even recyclable SERS systems [28]. However, these are expensive to synthesize and unsuitable for commercial use as easy, inexpensive synthesis is crucial for these substrates. Graphene, titanium, or silicon oxides, molybdenum disulfide, metal nanoparticles, and composites are candidate material systems [29,30,31]. However, metal nanoparticles remain the main material for the preparation of SERS substrates, largely since they allow the possibility of modifying the particle surface [32]. There are various ways to obtain metal nanoparticles. The main production method is the production of colloids by reduction from their salts [33]. However, there are other ways to obtain colloids, for example, obtaining particles by laser ablation [34]. This method makes it possible not to stabilize the nanoparticles with ligands. However, the resulting particles have a larger spread in size, which can worsen the SERS signal. The citrate method for obtaining gold nanoparticles, which is presented in this work, is the best choice. Because there is no need to add stabilizing agents, since the citrate ion not only reduces gold during the reaction, but also stabilizes the colloid nanoparticles, and the citrate ion is easily replaced by any desired ligands if necessary. Many synthesis techniques exist, including synthesis directly on the substrate [35] and the synthesis of a self-assembling film and the study of its properties in a solvent medium [36] deposited by thermal or electron beam evaporation onto a structured substrate [25,26,37].

The liquid–liquid interface provides a molecularly flat, defect-correcting platform for nanoparticle self-assembly. In addition, the obtained self-assembling films can rearrange when changing the shape of the substrate. Thus, self-assembled films of plasmonic nanoparticles (SPF) can be organized in cuvettes or test tubes and on flat substrates. There are methods to control the distance between particles during self-assembly. The use of self-assembling agents with different molecular sizes or a change in the ionic strength in the aqueous phase during self-assembly leads to a change in the packing density [38,39,40]. Some self-assembling systems are considered as enhancing media in liquid analyte solutions [36,41]. However, they are not suitable for commercial purposes, since they require synthesis directly before the measurement is made.

In this work, we consider SERS substrates based on SPF in a polymer matrix. In this study, SPF self-assembly parameters such as the size of gold nanoparticles, the type of the self-assembling agent, and its concentration in the non-polar self-assembly phase were optimized. Polystyrene, PVA, and polyethylene are considered as polymer matrices. The reproducibility and enhancement parameters of the resulting SERS substrates were evaluated. In addition, the minimum detectable concentration for the best sample was determined.

## 2. Materials and Methods

### 2.1. Materials

Chloroauric acid (99.995%); trisodium citrate; tetraoctylammonium bromide (TOABr); tetrabutylammonium nitrate (TBAN); 1-dodecanethiol (DDT); pseudoisocyanine iodide (97%); polystyrene M_w_ 35,000; M_w_ 192,000; poly(vinyl alcohol) with M_w_ 89,000–98,000, M_w_ 146,000–186,00, and M_w_ 13,000–23,000 were purchased from Aldrich. Toluene potassium hydroxide, hydrochloric acid, and nitric acid were purchased from Vekton, Russia. All chemicals were used without additional purification. Ultrapure water (Milli-Q) was used throughout the experiments.

Organic and inorganic contaminants were removed from the glassware before the syntheses. All glassware was washed in ethanolic potassium hydroxide to remove any organic impurities and then washed with water and further cleaned with aqua regia. Finally, the glass was thoroughly rinsed twenty times with deionized water.

### 2.2. Characterization

Raman spectra were recorded using a Renishaw inVia micro-Raman spectrometer, England, UK, with the 488 nm line of an Ar+ laser in backscattering geometry using a 50× Leica objective lens with an NA = 0.78.

SEM images were obtained using a Merlin-Zeiss microscope at 15 kV and a cathode current I = 5000 pA. An Oxford XMax 80 energy dispersive analysis module and Aztec software were used for elemental analysis.

A Solver Pro-M atomic-force microscope (AFM), supplied by NT-MDT, Russia, was used to study the morphology and thickness of the thin films.

Absorption spectra were recorded on a UV-3600 spectrophotometer from Shimadzu, Japan, in the range 300 nm to 800 nm.

We used the dynamic light scattering (DLS) method to determine the size of gold nanoparticles. The hydrodynamic size distribution and zeta potential of Au nanoparticles in solution were measured using a Malvern Zetasizer Nano from Malvern, England, UK.

### 2.3. Preparation and Characterization of Gold Colloids

**Figure 1:**
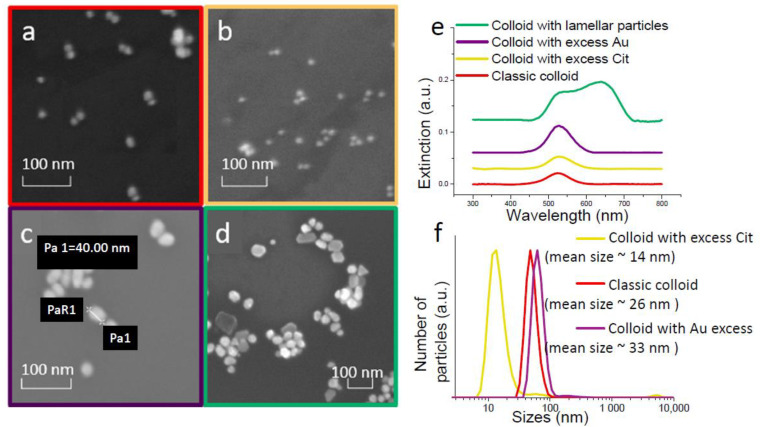
SEM images (**a**) colloid’s gold particles obtained by the classical method; (**b**) colloid’s gold particles obtained in trisodium citrate excess; (**c**) colloid’s gold particles obtained in excess chloroauric acid; (**d**) colloid’s gold particles with cooling immediately after synthesis of gold colloid; (**e**) absorption spectra of colloids; and (**f**) DLS results for colloids obtained by the classical method, in trisodium citrate excess, in chloroauric acid excess.

Gold nanoparticles were prepared by a citrate-reduction method, involving the reduction of metallic gold from chloroauric acid with sodium citrate [33]. Different concentrations of sodium citrate in the reaction mixture were used in the synthesis to obtain gold particles of different sizes. An increase in the concentration of sodium citrate in the reaction mixture leads to a decrease in the particle size, which was shown in [42]. Presumably, there is an increase in the number of nuclei and faster adsorption of citrate ion on the surface of gold particles with an excess of citrate ions. As a result, smaller Au nanoparticles were synthesized. During the reaction, the color changes from yellow to blue, then wine red. Three samples (Figure 1a–c) continued to heat up after the color change for an hour. Nanoparticles in these colloids have a spherical shape. For one sample, the reaction was slowed down by cooling immediately after the color change in the reaction mixture. A SEM picture of this sample of colloid nanoparticles (Figure 1d) shows that the particles have a lamellar form with an approximate size of 60 × 40 × 20 nm.

SEM images (Figure 1a–d), absorption spectra (Figure 1e) and DLS study results (Figure 1f) are shown in Figure 1. Varying the concentration of sodium citrate allowed us to obtain particles of various sizes. DLS are measured for three table colloids with spherical nanoparticles. The approximate particle size for a colloid obtained by the citrate-reduction method without changing the reagent concentrations, the classic method, is 26 nm. The approximate particle size in colloids obtained using an excess of sodium citrate is 14 nm. The approximate size of the colloid particle for the reaction mixtures that contained an excess of gold is 33 nm. These values are consistent with SEM data, which estimate the particle sizes to be 20 nm, 10 nm, and 40 nm for these three cases. Spectra from these samples have maxima close to ~530 nm, with a slight shift apparent as the particle size increases [41].

The colloid with lamellar nanoparticles turned out to be unstable during storage. Therefore, it was not possible to determine the size of the colloid by the DLS method. However, all samples necessary for the study were obtained from a freshly prepared colloid. Two maxima are observed in the absorption spectrum of this colloid at ~530 and ~638 nm. This is related to the plate shape of the colloid particles [43].

### 2.4. Preparation of SPF

**Figure 2:**
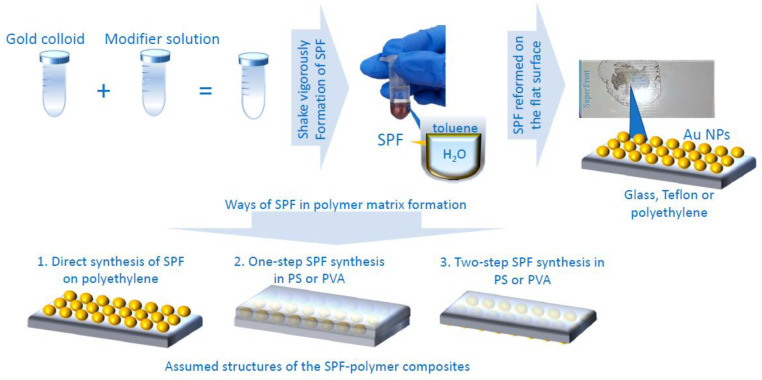
Synthesis scheme of SPF and approximate structure of SPF obtained in polymer matrix.

The synthesis procedure for the SPF is simple. The SPF synthesis scheme is shown in Figure 2. Gold colloid was vigorously shaken with a toluene solution of the modifier. Subsequently, the mixture was poured onto various substrates. The modifier is a self-assembling agent that changes the hydrophobicity of the surface of the colloid gold particles, and they form an SPF at the water–toluene phase boundary. In this work, three modifiers: TOABr, TBAN, and DDT were studied. The SPF were synthesized on glass, polytetrafluoroethylene (Teflon), and polymer substrates.

### 2.5. Preparation of Film in a Polymer Matrix Samples

Three techniques have been used to obtain films in a polymer matrix. Synthesis techniques for SPF in a polymer matrix are shown in Figure 2.

The first method is the synthesis of an SPF over a polymer. A commonly available household polyethylene film was used for this method. The polyethylene film was attached to a Petri dish, then SPF from a test tube was poured onto a polymer substrate. There were holes present in the polyethylene film, through which the reaction solution of water and toluene flowed. In this case, an island film was obtained on the polyethylene surface. This method is called direct synthesis of SPF in polyethylene (SPF-PE). The approximate structure of this SPF-PE film is shown in Figure 2 (1. Direct synthesis of SPF on polyethylene).

The second method of producing a film in a polymer matrix involved the simultaneous formation of the film and a polymer film. Polystyrene (PS) and polyvinyl alcohol (PVA) were chosen as polymer matrices for these SERS substrates. Various concentrations of all polymers were investigated to determine the thickness of the polymer layer at which it still remains possible to separate it from the synthesis substrate and changes in enhancement are still insignificant. In this method, the polymer was dissolved in one of the components of the solvent mixtures during film preparation. PS was dissolved in a toluene solution of the modifier. PVA was dissolved in an aqueous colloid of gold particles. This method of producing films was called one-step SPF synthesis in PS (SPF-PS-1) and PVA (SPF-PVA-1). The proposed structure of the SPF-PS-1 and SPF-PVA-1 is shown in Figure 2 (2. One-step SPF synthesis in PS and PVA).

The third way to synthesize a film in a polymer matrix is to obtain an SPF in a polymer matrix in two steps. The first step is the formation of an SPF on different substrates, then a polymer film is formed above the SPF. This method is called two-step synthesis of SPF in PS (SPF-PS-2) and SPF in PVA (SPF-PVA-2). The proposed structure of the SPF-PS-2 and SPF-PVA-2 is also shown in Figure 2 (3. Two-step SPF synthesis in PS and PVA).

### 2.6. Analyte Used in the Study

**Figure 3:**
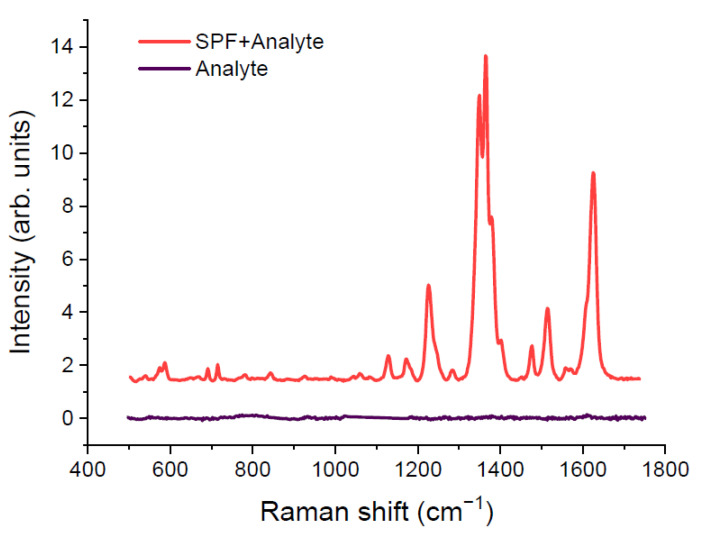
Comparison of analyte Raman spectrum and SERS spectrum. Dark violet line is analyte Raman spectrum, red line is SERS spectrum of analyte. Analyte concentration was equal 7.5 × 10^−6^ mol/L.

Pseudoisocyanine iodide was chosen as the analyte. The minimum detectable analyte concentration without enhancement was chosen to compare similar SERS substrates. This concentration is 7.50 × 10^−7^ M. Figure 3 shows the spectra of an analyte with a concentration of 7.50 × 10^−7^ M with enhancement (SPF + analyte) and without enhancement (analyte). The spectrum exhibits the most intense Raman band at 1364 cm^−1^ [44,45]. This band was chosen to compare the enhancement efficiency of SERS substrates based on SPF. The control experiment was performed for a concentration of 7.50 × 10^−6^ M. The increase in concentration makes it easier to determine the location of the band in the resulting Raman spectrum of the dye (analyte).

### 2.7. Calculation of Enhancement and Reproducibility Parameters

Using the well-known formula [4] (Formula (1)), a value proportional to the analytical enhancement factor (AEF) was employed in this work to compare similar SERS substrates.

(1) $$AEF=\left(I_{SERS}/I_{R}\right)\cdot \left(c_{R}/c_{SERS}\right)$$

where $I_{SERS}$ and $I_{R}$ are the intensities of the SERS and Raman signals, respectively, and $c_{SERS}$ and $c_{R}$ are the concentrations of the analyte in the SERS and control Raman experiments, respectively. In this formula, provided that one control experiment and one concentration are used, only the intensity changes. Thus, it is sufficient to compare only the intensities obtained from the SERS samples.

The intensity distribution map (IDM) over the surface of each SERS substrate was obtained for comparison of samples. The IDM has an area of 1600 μm^2^ and includes 81 spectra. The mapped area of the SPF samples was determined randomly but was not located at the edges of the area occupied by the SPF in order to avoid measurement-related errors. The average intensity of map (AIM) was calculated for the IDM data. The AIM was calculated for the most intense band of the Raman spectrum from the analyte at 1364 cm^−1^.

The reproducibility of the intensity in the IDM is proportional to the map data gate (MG). The MG was calculated by discarding 20% of the maximum and minimum values of the IDM data intensity. The MG is convenient for displaying on charts. However, the value of the percentage map gate (%MG) turned out to be a more useful indicator. The %MG was calculated by Formula (2):

(2) %MG =(MG/AIM)·100%

where MG is the data map gate and AIM is the average intensity of the samples map. The value of the %MG was useful in [46]. The %MG was used when comparing the reproducibility of signals from SERS substrates in a polymer matrix.

## 3. Results and Discussion

### 3.1. Determination of Optimal Parameters for SPF Synthesis

The preparation of films based on SPF in a polymer matrix requires the determination of the concentrations of the reagents for the SPF synthesis. The parameters proportional to the enhancement factor and reproducibility (Section 2.7) of the substrate SERS signal were calculated to determine an optimal SERS substrate. The calculation of these parameters was carried out according to the data obtained during the mapping of the SERS substrates based on SPF. The varied SPF synthesis parameters were the type of the modifier (DDT, TOABr, TBAN), the modifier concentration, and gold particle size. Previously, a study was carried out where it was shown that the SPF structure, which gives the maximum SERS signal, corresponds to a certain optimal ratio of the concentrations of gold nanoparticles and modifier [47].

**Figure 4:**
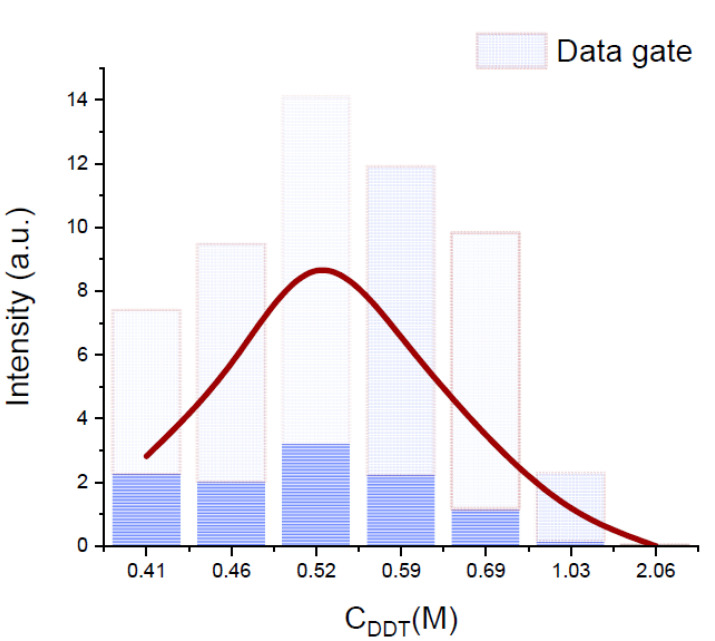
Intensity distribution of 1364 cm^−1^ band in spectral map of samples with 1-dodecanethiol as self-assembling agent. The typical form of the dependence of the intensity on the concentration of the modifier for all studied modifiers. Boxes indicate the SERS intensity distribution of the gain data.

All dependencies of the average intensity of map (AIM) vs. concentrations have a maximum. The concentration corresponding to the maximum AIM is referred to as the optimal concentration. The dependence of the signal intensity on the concentration of the DDT modifier is shown in Figure 4. The AIM values by intensity are connected by a smooth curve. The rectangles in the histogram show the MG values. The maximum value of AIM is observed at C(DDT) = 0.52 M. This concentration is much higher than the optimal concentration of TBAN and TOABr. SPF synthesized with DDT as a modifier are of poor quality. The SPF islands formed an increase in height but do not increase in planar extent. As a result, they do not coalesce to form a continuous film. This leads to an uneven enhancement efficiency and a poor reproducibility of the enhancement factor over the substrate surface. Therefore, it was decided to abandon the study of samples with this modifier.

**Figure 5:**
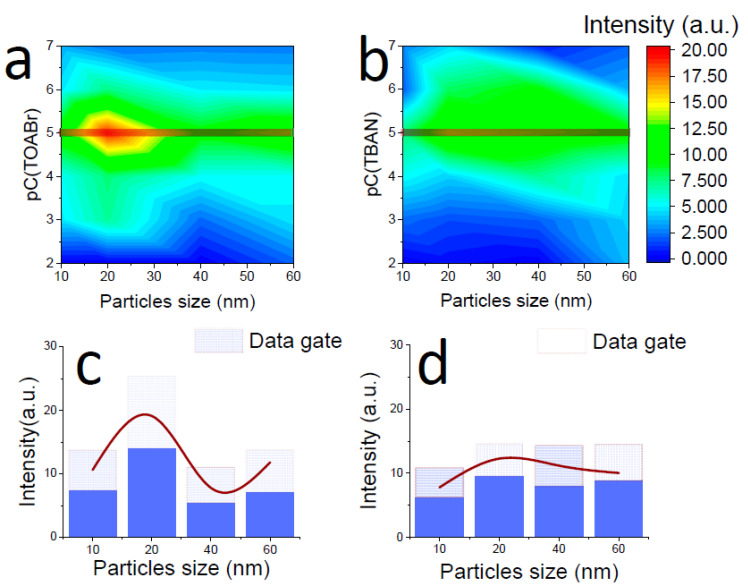
(**a**) Average intensity of 1364 cm^−1^ band in spectral map distribution for different particle sizes and concentrations of TOABr as self-assembling agent. Red color is maximum of average intensity of spectral map; (**b**) Average intensity of 1364 cm^−1^ band in spectral map distribution for different particle sizes and concentrations of TBAN as self-assembling agent; (**c**) Intensity distribution of 1364 cm^−1^ band in spectral map of samples with TOABr as self-assembling agent for different sizes of gold nanoparticles in SPF; and (**d**) Intensity distribution of 1364 cm^−1^ band in spectral map of samples with TBAN as self-assembling agent for different sizes of gold nanoparticles in SPF. Boxes indicate the SERS intensity distribution of the gain data.

Calculated dependencies for SPF samples with TBAN and TOABr as a modifier are shown in Figure 5. Three-dimensional maps were obtained for comparing data from samples of SERS substrates for which TOABr and TBAN were used as modifiers in the synthesis of SPF (Figure 5a,b). The color indicates the AIM values that correspond to this point on the map. AIM values have a range from 0.05 to 20 arb units. The *X*-axis shows values of the size of the gold particles in the colloids used in the synthesis of the SPF. Particles 10, 20, and 40 nm in size were spherical in shape. Lamellar gold particles with dimensions of 60 × 40 × 20 nm were included in the Figure. A sample of a film of lamellar particles is designated as 60 nm for ease of plotting. The *Y*-axis shows the concentration of the modifier in the organic solution. The concentration of modifiers (TBAN, TOABr) in the figures is given using a logarithmic scale multiplied by −1, that is, pC = −log_10_(C). The map shows that the maximum AIM values for both modifiers correspond to a concentration of 10^−5^ M. The dependencies of the AIM on the size of the gold particles of the colloid used in the synthesis of the SPF are shown under the maps in Figure 5c,d. The smooth curve shows the dependence of the AIM on gold particle size. The MG of all SPF samples are shown by rectangles in histograms. A comparison of SPF samples with different size particles revealed that the maximum AIM value corresponds to the SPF sample with 20 nm particles. The 60 nm SPF sample had higher AIM values than the 40 nm SPF sample. However, the lamellar shape of the particles did not affect the AIM value and the shape of the intensity–concentration curve. These samples are included in the consideration, along with other SPF samples.

SERS substrates must have reproducible enhancement factors on their surface. This is important for routine SERS studies since the site of the analyte deposition on the SERS substrate should not affect the efficiency of the analyte signal enhancement. The %MG values are shown in Table 1. The minimum %MG value represents the best reproducibility of the SERS signal over the surface of the SPF of the samples. An SPF sample prepared from 20 nm gold particles and TOABr solution has a minimum %MG = 28%. A good reproducibility of the SERS signal depends on the distribution of hot spots. In our case, we assume that the main type of hot spots is the interparticle type. Thus, the packing density of the resulting SPF is of the greatest importance for signal reproducibility. Therefore, we can assume that for an SPF sample with a nanoparticle size of 20 nm and TOABr as a modifier, the most favorable dense packing is realized, which provides the maximum SERS response and the best reproducibility.

The morphology of SPF samples was studied for two modifiers, TOABr and TBAN. Synthesis parameters were selected based on the maximum AIM values.

### 3.2. Characterization of the Morphology of the SPF Samples with Maximum Values for the Average Map Intensity

**Figure 6:**
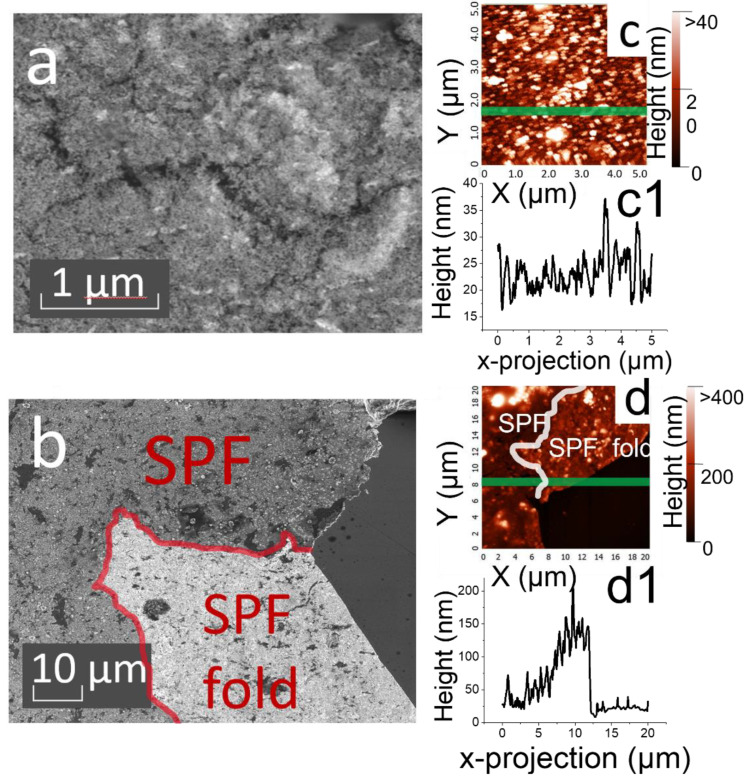
(**a**) SEM image of SPF with TBAN as self-assembling agent; (**b**) SEM image of SPF with TOABr as self-assembling agent; (**c**) AFM image of SPF with TBAN as self-assembling agent ((**c1**)—surface profile of the SPF sample at Y = 1.7 μm); and (**d**) AFM image of SPF with TOABr as self-assembling agent ((**d1**)—surface profile of the SPF sample at Y = 15 μm).

SEM and AFM studies were carried out for films with maximum AIM values. SEM images of the film are shown in Figure 6a. Firstly, it is necessary to describe the morphology obtained with the addition of TBAN solution as an SPF modifier. The resulting SPF consists of a larger number of islands. This was observed visually during the synthesis of the SERS substrate on glass. The SEM images show the formation of film islands without characteristic features, such as the SPF sample synthesized with a TOABr solution. AFM studies reveal further details of the film thickness and morphology. Figure 6c shows images from the middle of the sample obtained with the addition of TBAN SPF. The film in the middle possesses a more uniform thickness distribution, with an average thickness of about 50 nm. Figure 6c1 shows the surface profile of the SPF sample at Y = 1.7 μm. Considering the approximate multiplicity of the average size of the film at the edge, we assume that in the central part the film is formed by a mono- or bilayer with inclusions of particle aggregates.

The features of the morphology of the SPF obtained with the addition of TOABr solution as a modifier are now described. The optimal SPF has “folds” at the edges of the SPF (Figure 6b). This can be seen as a difference in color between the top and bottom of the SPF. This indicates that the TOABr is incorporated by the hydrocarbon part into the organic part during synthesis and is located in the upper part of the SPF after formation of the SERS substrate. A similar edge of the SPF was studied by AFM (Figure 6d). According to the data obtained with the AFM, it is obvious that the film includes not only gold particles, but also their aggregates, which were seen when studying SEM images of colloids (Figure 1). Figure 6d1 shows the surface profile of the SPF sample at Y = 8 μm. This line crosses the edge of the SPF, making it possible to assert that the film consists of several layers.

The optimal size for obtaining the SERS substrate was found to be 20 nm. A colloid with this particle size will be used to obtain the SPF in polymer matrices. The concentration of the modifier in the toluene phase during the synthesis of the SPF for which maximum enhancement is observed is 10^−5^ M. The AIM values are 19.10 and 12.28 arb. units for TOABr and TBAN, respectively. Thus, the following parameters were chosen to obtain SPF in a polymer matrix: gold particles with a size of 20 nm and TOABr as the modifier with a concentration in the organic phase of 10^−5^ M.

### 3.3. Comparison of SERS Substrates Based on SPF in a Polymer Matrix

There are many ways to obtain SERS substrates. In our study, we considered substrates based on SPF in a polymer matrix. As described in paragraph 2.5 in the Experimental section, we used various methods to obtain substrates in polymer matrices in this study. Presumptive structural features depending on the synthesis are as shown in Figure 2. Polystyrene, polyvinyl alcohol, and polyethylene were chosen to describe the features of the formation of SERS substrates based on SPF in a polymer matrix. Polymers with different molecular weights were used in the synthesis of SPF in a polymer matrix. As expected, higher molecular weight polymers formed polymer films that were easily removed from the substrate. In contrast, low molecular weight polymer films crumbled during the separation from glass, since they did not form an easily removable elastic SERS substrate.

**Figure 7:**
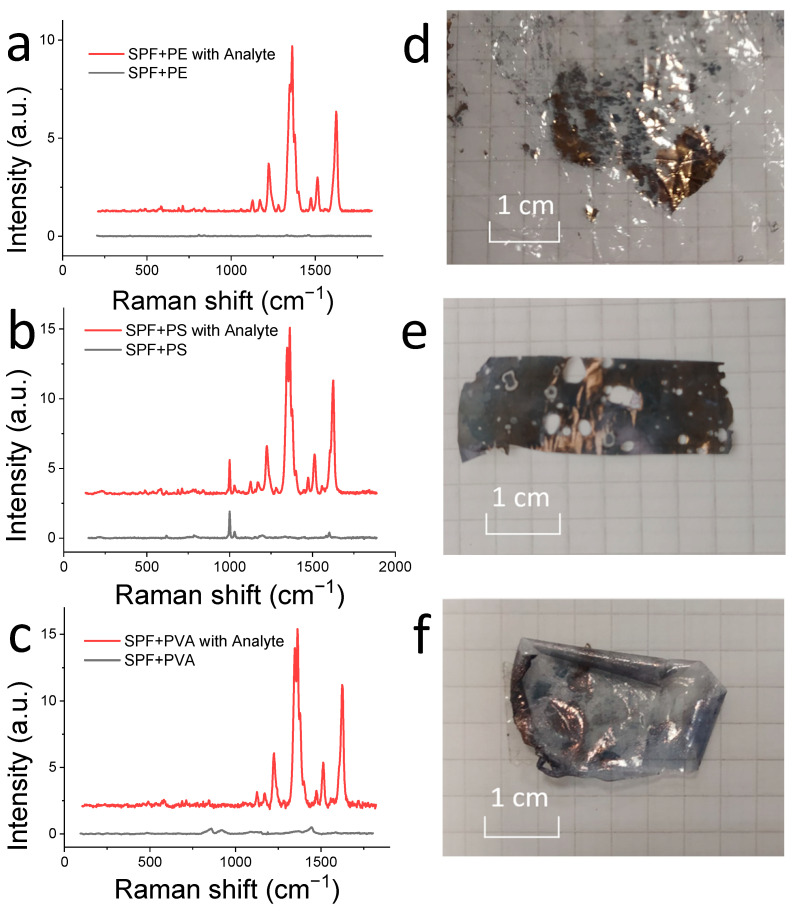
(**a**) Raman spectra of SPF-PE-1 sample without analyte (black), and with analyte (red); (**b**) Raman spectra of SPF-PS-1 sample without analyte (black), and with analyte (red); (**c**) Raman spectra of SPF-PVA-1 sample without analyte (black), and with analyte (red); (**d**) Photo of SPF-PE-1 sample; (**e**) Photo of SPF-PS-1 sample; and (**f**) Photo of SPF-PVA-1 sample.

Raman spectra of the SERS substrates, both without analyte and with applied analyte, are shown in Figure 7a–c. Due to the additive property of the spectra of mixtures, the spectra of the substrate can be subtracted from the analyte spectrum when using these SERS substrates. Therefore, before mapping the samples with the applied analyte, a “base map” was obtained from the sample without the analyte. Then, the analyte was applied to the sample and the registration and mapping of the Raman spectra of the analyte took place. Raman spectra with an enhanced analyte signal and base map are shown on the figures for clarity. The AIM and %MG values were calculated for the most intense band of the analyte Raman spectrum.

Raman spectra of the SERS substrates, both without analyte and with applied analyte, are shown in Figure 7a–c. Due to the additive property of the spectra of mixtures, the spectra of the substrate can be subtracted from the analyte spectrum when using these SERS substrates. Therefore, before mapping the samples with the applied analyte, a “base map” was obtained from the sample without the analyte. Then the analyte was applied to the sample and the registration and mapping of the Raman spectra of the analyte took place. Raman spectra with an enhanced analyte signal and base map are shown on the figures for clarity. The AIM and %MG values were calculated for the most intense band of the analyte Raman spectrum.

Photos of the SERS substrate samples obtained in different polymer matrices are shown in Figure 7d–f. Mapping of the SERS substrates, with a polymer matrix of PVA and PE, was hindered by the pronounced curvature of the sample surface, making focusing problematic. The PE had irregularities associated with the pores, through which solvents of the reaction mixture flowed down during the SPF synthesis. The curvature of the PVA samples is due to the fact that the polymer is sensitive to the humidity of the surrounding atmosphere and curls upon drying. The values of the parameters calculated to evaluate and compare the enhanced efficiency and reproducibility are shown in Table 2. Mapping was done on both sides for all samples. When obtaining close AIM values, it was assumed that the SPF is inside the polymer matrix. The approximate structure of these samples is shown in Figure 2(2). Obtaining different AIM values suggests that the SPF is under the polymer matrix. The approximate structure of these samples is shown in Figure 2(3). SPF-PS showed higher AIM values and similar values to the SPF-PVA and SPF-PE %MG samples.

The SPF-PS syntheses were carried out with a change of the substrate on which the SPF was synthesized. Glass and polytetrafluoroethylene (Teflon) were studied as substrates for synthesis. The AIM and %MG data for these samples are shown in Table 3. SPF-PS samples are more easily removed from Teflon during the experiment. However, when the SPF-PS-2 sample was removed, some of the SPF remained on the Teflon. Removal of the SPF from the glass/Teflon substrate leads to splitting of the SPF layers and better signal amplification, due to the fact that the SPF consists of several layers. Perhaps because of this, the side with the SPF of the SPF-PS-2 sample has higher AIM values and lower %MG values. This is only apparent when mapping without focus regulation during the experiment, but not when single spectra or small maps are obtained.

Thus, the SPF sample in the polymer matrix, which showed the maximum enhancement values and the best reproducibility parameters for pseudoisocyanine iodide, has the parameters:

- This is a sample obtained by the method of one-step synthesis;
- The concentration of polystyrene is 4.5 mg/mL and TOABr 10^−5^ mol/L in the toluene phase;
- The size of gold nanoparticles is 20 nm.

**Figure 8:**
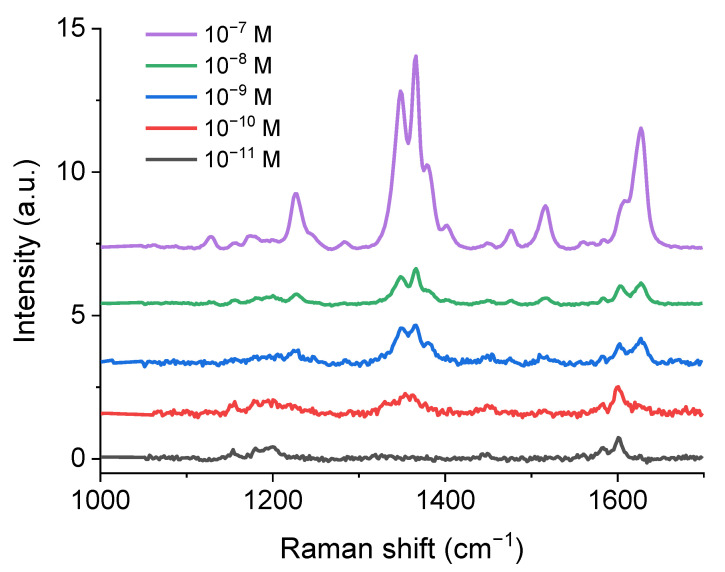
Raman spectra of SPF-PS-1 sample with different concentrations of analyte from 10^−7^ M to 10^−11^ M.

Finally, a study of the minimum detectable concentration of the analyte for the SPF-PS sample was carried out. Raman spectra for different analyte concentrations are shown in Figure 8. Analyte solutions with a concentration from 10^−7^ M to 10^−11^ M were applied to the SPF-PS sample. The minimum detectable concentration is 10^−10^ M. The analytical enhancement factor of the SERS substrate studied is 2.7 × 10^4^.

## 4. Conclusions

In conclusion, a study of the synthesis parameters of the SPF was performed. The influence of the size of the gold particles that make up the SPF was investigated. SPF samples with a gold particle size of 20 nm showed the best enhancement. Various modifiers for SPF self-assembly are studied and TOABr was judged to be best for SPF synthesis. The concentration at which films with higher SERS signal intensities are synthesized is 10^−5^ M.

Various polymers for the synthesis of SPF in a polymer matrix were investigated. SPFs were synthesized in a polymer matrix in three different ways. The direct method involves pouring the SPF synthesis mixture of solvents onto a polymer substrate. One-step synthesis is the simultaneous synthesis of SPF and polymer. Two-step synthesis involves the synthesis of the SPF, then the production of a polymer film on top of the SPF. The SPF-PVA samples were found to be sensitive to atmospheric humidity. SPF-PE showed low SERS signal values. One-step synthesis has an advantage over two-step synthesis in two respects. Firstly, the SERS signal is enhanced in the same way on both sides of the SERS substrate. Furthermore, the SPF synthesized by one-step synthesis is not subject to mechanical damage since it is located inside the polymer matrix. The minimum detectable concentration was measured on the SERS substrate obtained and found to be 10^−10^ M (0.454 ppb). The analytical enhancement factor of the studied SERS substrate is 2.7 × 10^4^.

## Figures and Tables

**Table 1:** %MG values of SPF samples with different size gold particles.

Modifier	Size of Gold Spherical Nanoparticles, nm	Percentage Data Gate (%MG), %
TOABr	10	62
TOABr	20	28
TOABr	40	72
TOABr	lamellar 60 × 40 × 20	87
TBAN	10	71
TBAN	20	49
TBAN	40	59
TBAN	lamellar 60 × 40 × 20	56

**Table 2:** AIM and %MG values of SPF in different polymer matrix samples.

Polymer Type	Type of Synthesis and Resulting Sample	Average Intensity (1364 cm^−1^) of Sample Map Data (AIM), a.u.	Percentage Data Gate (%MG), %
Polystyrene	One-step SPF synthesis; SPF-PS-1	6.84	30.84
	Two-step SPF synthesis; SPF-PS-2	2.11	90.18
	Two-step SPF synthesis; SPF-PS-2	8.10	91.33
Polyvinyl alcohol	One-step SPF synthesis; SPF-PVA-1	5.22	68.96
	Two-step SPF synthesis; SPF-PVA-2	5.14	94.54
	Two-step SPF synthesis; SPF-PVA-2	2.09	65.54
Polyethylene	Direct synthesis; SPF-PE	3.35	37.44
	Direct synthesis; SPF-PE	0.52	153

**Table 3:** AIM and %MG values of synthesis of SPF-PS on different substrates.

SPF Synthesis Substrate	Average Intensity (1364 cm^−1^) of Sample Map Data (AIM), a.u.	Percentage Data Gate (%MG), %
Glass	5.22	68.96
Glass Au-top side	5.14	94.54
Glass Au-down side	2.09	65.54
Teflon	7.82	41.71
Teflon Au-down side	1.65	64.98
Teflon Au-top side	9.22	27.92
